# Periprocedural Antithrombotic Treatment During Acute Mechanical Thrombectomy for Ischemic Stroke: A Systematic Review

**DOI:** 10.3389/fneur.2018.00238

**Published:** 2018-04-16

**Authors:** Rob A. van de Graaf, Vicky Chalos, Gregory J. del Zoppo, Aad van der Lugt, Diederik W. J. Dippel, Bob Roozenbeek

**Affiliations:** ^1^Department of Neurology, Erasmus MC University Medical Center, Rotterdam, Netherlands; ^2^Department of Radiology, Erasmus MC University Medical Center, Rotterdam, Netherlands; ^3^Department of Public Health, Erasmus MC University Medical Center, Rotterdam, Netherlands; ^4^Division of Hematology, Department of Medicine, University of Washington School of Medicine, Seattle, WA, United States; ^5^Department of Neurology, University of Washington School of Medicine, Seattle, WA, United States

**Keywords:** ischemic stroke, periprocedural, heparin, antiplatelet agents, antithrombotic agents, mechanical thrombectomy, endovascular treatment

## Abstract

**Background:**

More than one-third of the patients with ischemic stroke caused by an intracranial large vessel occlusion do not recover to functional independence despite fast and successful recanalization by acute mechanical thrombectomy (MT). This may partially be explained by incomplete microvascular reperfusion. Some antithrombotics, e.g., antiplatelet agents and heparin, may be able to restore microvascular reperfusion. However, antithrombotics may also increase the risk of symptomatic intracranial hemorrhage (sICH). The aim of this review was to assess the potential safety and functional outcome of periprocedural antiplatelet or heparin use during acute MT for ischemic stroke.

**Methods:**

We systematically searched *PubMed, Embase, Medline, Web of Science*, and *Cochrane* for studies investigating the safety and functional outcome of periprocedural antiplatelet or heparin treatment during acute MT for ischemic stroke. The primary outcome was the risk for sICH. Secondary outcomes were functional independence after 3–6 months (modified Rankin Scale 0–2) and mortality within 6 months.

**Results:**

837 studies were identified through the search, of which 19 studies were included. The sICH risks of the periprocedural use of antiplatelets ranged from 6 to 17%, and for heparin from 5 to 12%. Two of four studies reporting relative effects of the use of antithrombotics are pointing toward an increased risk of sICH. Among patients treated with antiplatelet agents, functional independence varied from 23 to 60% and mortality from 18 to 33%. For heparin, this was, respectively, 19–54% and 19–33%. The three studies presenting relative effects of antiplatelets on functional independence showed neutral effects. Both studies reporting relative effects of heparin on functional independence found it to increase this chance.

**Conclusion:**

Randomized controlled trials investigating the effect of periprocedural antithrombotic treatment in MT are lacking. Some observational studies report a slight increase in sICH risk, which may be acceptable because they also suggest a beneficial effect on functional outcome. Therefore, randomized controlled trials are warranted to address the question whether the potentially higher risk of sICH could be outweighed by improved functional outcome.

## Background

The introduction of endovascular treatment by means of acute mechanical thrombectomy (MT) has been a major change in the emergent treatment of ischemic stroke caused by an intracranial large vessel occlusion. An individual patient data meta-analysis of randomized trials showed that this approach is highly effective (1). In that meta-analysis, MT significantly improved functional outcome at 90 days, with a number needed to treat of 2.6 to reduce disability by one level on the modified Rankin Scale (mRS). Still, approximately one-third of the patients do not recover to functional independence despite fast and complete recanalization by MT (2, 3). This could partially be attributable to microvascular dysfunction also known as incomplete microvascular reperfusion (IMR). The concept of IMR stems from observations in the non-human primate of focal “no-reflow” following focal ischemia—caused by adhesion of polymorphonuclear leukocytes (4–6), and/or platelet-fibrin occlusions (7) within the downstream microvasculature—that could be prevented by anti-leukocyte or antithrombotic strategies. More recently, this concept has been described again (8). Antiplatelet agents in experimental systems have shown to prevent the microvascular occlusive events in both non-human primate and mouse models and to improve outcome (9, 10). Also heparin may be of additional value to MT, by preventing microthrombus formation and microvascular obstruction and potentially restore microvascular reperfusion. It has been suggested that microvascular obstructions could arise from neutrophil extracellular trap (NET) formation (11). NET formation can be dissolved by heparin, but not by tissue plasminogen activator (tPA) (12, 13). As antiplatelet agents and heparin seem promising in their ability to restore microvascular function, these drugs might contribute to the recovery of patients with ischemic stroke undergoing acute MT. A direct test of this hypothesis in humans has not yet taken place. An important disadvantage of both antiplatelet and heparin use in the setting of focal cerebral ischemia is the increased risk of intracranial hemorrhage. Symptomatic intracranial hemorrhage (sICH) leads to severe handicap or death in almost all patients (14). A randomized trial—in which patients with an ischemic stroke were either assigned to intravenous (IV) antiplatelet agents within 90 min after starting treatment with IV recombinant tPA or to no antiplatelet agents—was stopped before the intended conclusion due to non-superior outcomes and a higher risk of sICH in the group that received antiplatelet agents (15). Although the absolute sICH risk associated with acute antiplatelet administration was low (4.3%), concerns remain about this detrimental side effect. These concerns are also present with regard to the use of heparin in ischemic stroke. This may be due to the results of the International Stroke Trial, in which 19,435 patients were randomized to receive antiplatelet agents, heparin, both or neither within 48 h after symptom onset (16). In this study, the beneficial results (i.e., reduced risk of recurrent stroke and improved functional outcome) were offset by a higher sICH risk. Again, the absolute sICH risk was low in this trial, even in the high-dose group receiving 12,500 IU twice daily (2.0%). Yet, the balance between risk and benefit of these antithrombotic drugs for patients with ischemic stroke is uncertain in the setting of acute MT. Therefore, the aim of this review was to assess the potential safety and functional outcome of periprocedural antiplatelet or heparin use during acute MT for ischemic stroke.

## Methods

### Search Strategy

A search strategy was developed in collaboration with a biomedical information specialist to systematically search *PubMed, Embase, Medline, Web of Science*, and *Cochrane*. The search was conducted in November 2017 and updated in March 2018. Two independent reviewers (RG and VC) screened all identified articles on titles and abstracts for eligibility. Articles identified as potentially eligible underwent a full text review. Disagreements between reviewers were resolved by a consensus meeting with a third reviewer (BR). The complete search strategy is listed as supplemental material (Data Sheet S1 in Supplementary Material).

### Inclusion and Exclusion Criteria

Studies were eligible for inclusion when:

– Periprocedural [consisting of prior, *acute* (<6 h) or *early* (6–24 h)], oral or parenteral, antiplatelet agents or heparin were used in patients who underwent MT for ischemic stroke.
– Posttreatment sICH was reported.
– English abstract was available.
– Patients were 18 years or older.

Studies were excluded when:

– Antithrombotic agents other than antiplatelet agents and heparin were used.
– The specific number of patients with prior antiplatelet agents could not be extracted, and differentiation between outcomes of patients with and without prior antiplatelet use was not possible.
– Less than 50% of the endovascular treated patients were treated with MT.
– Less than 20 patients underwent MT.

In addition, studies reporting on patients with “tandem lesions” (i.e., an intracranial large vessel occlusion with simultaneous ipsilateral extracranial carotid occlusion) treated with intracranial MT with or without emergency carotid artery stenting were included through bibliographic review of the included studies. In these studies, antithrombotics were used as a part of protocol-based care to prevent stent occlusion. Finally, large randomized controlled trials (RCTs) investigating the effectiveness of MT, and in which periprocedural antithrombotics were used, were included through bibliographic reviewing.

### Data Extraction and Synthesis

We developed a data extraction form based on elements of the Cochrane Consumers and Communication Review Group’s data extraction template (17). Two reviewers extracted the data independently: one reviewer extracted all the data (RG) and the other reviewer extracted 25% of the data (VC). Extracted data were checked during consensus meetings with three reviewers (RG, VC, and BR). For each included study, we aimed to specifically extract the available data for the patients treated with MT or the most representative group. The following information was extracted: study design, study population characteristics [sample size, age, National Institutes of Health Stroke Scale (NIHSS) at baseline, and occlusion location], recanalization therapy [administration of IV plasminogen activators, administration of intraarterial (IA) plasminogen activators, treatment with MT, and time from symptom onset to recanalization therapy]; study treatment and contrast [type of antithrombotic treatment, indication for antithrombotic administration, time from symptom onset to antithrombotic treatment, number of patients treated with antithrombotic treatment, and information about the control group (when available)]; safety (sICH and all-cause mortality within 6 months); and functional outcome (functional independence after 3–6 months, expressed as a mRS score of 0–2) (18, 19). Special note was made of the definition of sICH in each study.

When available, study characteristics were reported by mean (SDs) or median (interquartile ranges). Outcomes were reported as numbers of cases and percentages. When a comparison was performed or a contingency table could be prepared, odds ratios for both safety (sICH and all-cause mortality) and functional (mRS 0–2) outcomes were reported, with 95% confidence intervals (CIs). If present, adjusted odds ratios (aORs) were also reported. When data were unclear or missing, we extracted data from the related original study (when available) or approached the corresponding author for clarification. Data were reported according to the Preferred Reporting Items for Systematic reviews and Meta-analyses (PRISMA) Statement (20). The checklist can be found in the supplementary material (Data Sheet S2 in Supplementary Material).

## Results

### Study Selection

**Figure 1:**
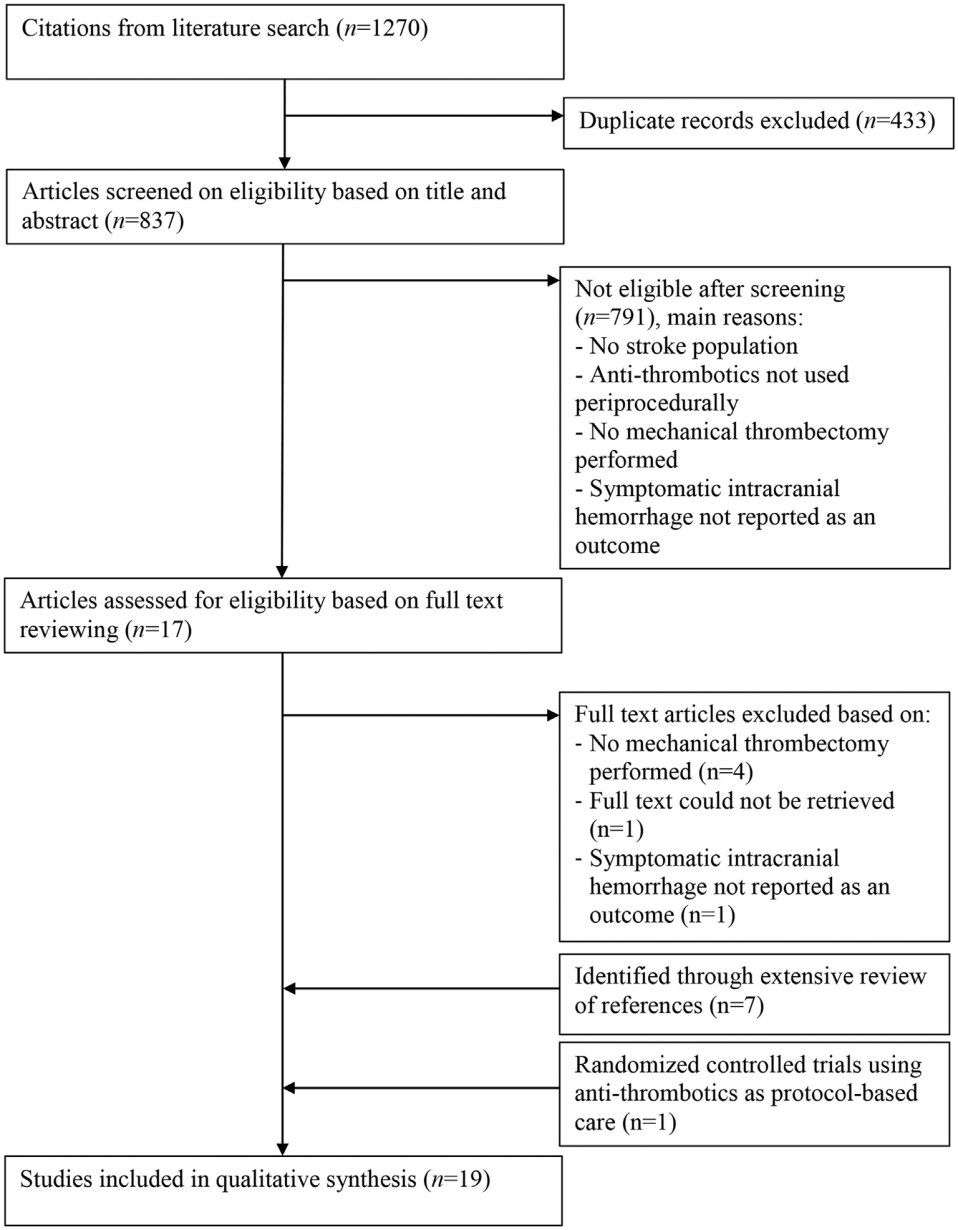
Flowchart of the systematic literature search.

The systematic literature search yielded a total of 1,270 studies (Figure 1). After removing duplicates, 837 articles remained, of which all titles and abstracts were screened. Full text of 17 articles was retrieved and assessed for eligibility. In addition, eight eligible studies were identified through bibliographic review of the included studies. Seven studies were identified in which tandem lesions were investigated, and one RCT investigating the effectiveness of MT was identified, in which periprocedural antithrombotics were used. A total of 19 articles met the selection criteria and were included in the review (21–39).

### Thrombectomy and *Antiplatelet Use*

We identified six studies investigating the periprocedural use of antiplatelet agents (22, 24, 31, 32, 36, 39). These studies include five cohort studies with sample sizes between 35 and 231 patients (22, 24, 31, 36, 39), and one *post hoc* analysis on a phase III RCT of 233 patients (Table 1) (32). The occlusion location varied between anterior circulation only (one study) (32), posterior circulation only (one study) (24), and both anterior and posterior circulation (three studies) (22, 36, 39). The occlusion location could not be retrieved in one study (31). In the cohort studies, 57–100% of the study population underwent MT, and in the *post hoc* analysis on phase III RCT data, all patients (in whom the effect of antiplatelet agents was investigated) underwent MT. The indication for antiplatelet use was mainly based on comorbidity (prior use) and prevention of re-occlusion of the vessel after recanalization. The sICH risk for periprocedural antiplatelet use ranged from 6 to 17%. Among the patients using antiplatelet agents, mortality varied from 18 to 33%, and functional independence from 23% to 60% (Table 2).

**Table 1:** Characteristics of included studies investigating periprocedural antiplatelet use in patients with ischemic stroke who underwent acute MT.

Study characteristics		Population characteristics				Recanalization therapy				Study treatment and contrast					
			
Reference	Study design	*N*	Age	NIHSS score at baseline	Occlusion location	IV tPA, *n*/*N* (%)	IA tPA,*n*/*N* (%)	MT, *n*/*N* (%)	Time from symptom onset to recanalization therapy (min)	Antithrombotic treatment	Indication for antithrombotic treatment	Time from symptom onset to antithrombotic treatment^a^	Treatment, *n*/*N* (%)	Control	Control, *n*/*N* (%)
Broeg-Morvay et al.^a^ (22)	Prospective cohort	231^b^	Mean^c^: 69 (± 14)	Median^c^: 15 (2–37)	Anterior + posterior circulation	231/231 (100%)	69/231 (30%)	212/231 (92%)	Mean^c^: 270 (±83)	ASA loading dose (median: 300 mg)	Prevention of re-occlusion Stenting	Acute	50/231 (22%)	No ASA	181/231 (78%)

Ernst et al. (24)	Retrospective cohort	54^b^	Mean: 65	Median: 32	Posterior circulation	0/54 (0%)	54/54 (100%)	31/54 (57%)	Median: 198	IV abciximab bolus (0.25 mg/kg) followed by continuous infusion, or, tirofiban bolus (10 μg/kg) followed by continuous infusion	Protocol-based care	Acute	54/54 (100%)	NR	NR

Memon et al. (31)	Prospective cohort	35^b^	Mean: 62	Median: 13 (5–22)	NR	2/35 (6%)	12/35 (34%)	≥20/35 (≥57%)	Median: 230	IA eptifibatide bolus (180 μg/kg)	Presence of distal emboli Inaccessible location by MT Prevention of re-occlusion	Acute	35/35 (100%)	NR	NR

Mulder et al. (32)	*Post hoc* analysis on phase III RCT	233	Median: 66 (55–76)	Median: 17 (14–21)	Anterior circulation	203/233 (87%)	24/233 (10%)	233/233 (100%)	Median: 260 (210–313)	Any antiplatelet use (single and dual)	Comorbidity	Prior use	64/233 (27%)	No antiplatelet use	169/233 (73%)

Pandhi et al. (39)	Retrospective cohort	217	Mean^c^: 60 (±14)	Median^c^: 16 (12–21)	Anterior + posterior circulation	141/217 (65%)	0/217 (0%)	217/217 (100%)	Mean: 361	Any antiplatelet use (single and dual)	Comorbidity	Prior use	71/217	No antiplatelet use	146/217 (67%)

Sugiura et al. (36)	Prospective cohort	204^b^	Mean: 71 (± 13)	Median: 18 (13–22)	Anterior + posterior circulation	80/204 (39%)	42/204 (21%)	170/204 (83%)	Mean: 188 (±101)	Any antiplatelet use (single and dual)	Comorbidity	Prior use	48/204 (24%)	No antiplatelet use	156/204 (76%)

*Characteristics of the included studies are presented by sample size (percentage), means (SDs), medians (interquartile ranges), or by remarks*.

*ASA, acetylsalicylic acid; IA, intraarterial; IV, intravenous; MT, mechanical thrombectomy (by means of stent retriever or aspiration); NIHSS, National Institutes of Health Stroke Scale; NR, not reported; RCT, randomized controlled trial; tPA, tissue plasminogen activator*.

*^a^Time from symptom onset to antithrombotic treatment was divided into acute treatment administration (<6 h) and early administration (6–24 h)*.

*^b^Not solely MT*.

*^c^Extracted data from the subgroup that received antiplatelet agents*.

**Table 2:** Outcomes of included studies investigating periprocedural antiplatelet use in patients with ischemic stroke who underwent acute mechanical thrombectomy.

Reference	sICH, *n*/*N* (%)	Mortality, *n*/*N* (%)	mRS (0–2), *n*/*N* (%)	sICH, OR (95% CI)	Mortality, OR (95% CI)	mRS (0–2), OR (95% CI)	sICH, aOR (95% CI)	Mortality, aOR (95% CI)	mRS (0–2), aOR (95% CI)
Broeg-Morvay et al. (22)	T = 3/50 (6%), C = 10/181 (6%)^b^	T = 9/50 (18%), C = 41/181 (23%)	T = 17/50 (34%), C = 83/181 (46%)	0.92 (0.24–3.46)	0.75 (0.34–1.67)	0.61 (0.32–1.17)	NR	NR	NR
Ernst et al. (24)	T = 7/54 (13%)^c^	T = 18/54 (33%)	T = 15/54 (28%)	NR	NR	NR	NR	NR	NR
Memon et al. (31)	T = 5/35 (14%)^d^	T = 8/35 (23%)	T = 21/35 (60%)	NR	NR	NR	NR	NR	NR
Mulder et al. (32)	T = 11/64 (17%), C = 7/169 (4%)^d^	T = 21/64 (33%), C = 28/169 (17%)	T = 15/64 (23%), C = 61/169 (36%)	4.80 (1.77–13.02)	2.46 (1.27–4.76)	0.54 (0.28–1.05)	NR	NR	NR
Pandhi et al. (39)	T = 4/71 (6%), C = 10/146 (7%)^e^	T = 18/71 (25%), C = 38/146 (26%)	T = 33/71 (50%), C = 64/146 (48%)	0.81 (0.25–2.68)	0.97 (0.50–1.85)	1.11 (0.63–1.97)	NR	NR	NR
Sugiura et al. (36)	T = 6/48 (13%), C = 4/156 (3%)^f^	NR	NR	5.43 (1.46–20.13)	NR	NR	8.03 (1.83–41.70)^a^	NR	NR

*aOR, (adjusted) odds ratio; C, control; CI, confidence interval; mRS, modified Rankin Scale; NR, not reported; sICH, symptomatic intracranial hemorrhage; T, treated with antiplatelet agent; NIHSS, National Institutes of Health Stroke Scale*.

*^a^Adjusted for glucose level and NIHSS at baseline*.

*^b^PROACT-II definition (40)*.

*^c^Intracranial hemorrhage resulting in NIHSS increase of >4 or non-definable neurologic status with PH and severe mass effect or subarachnoid hemorrhage with hydrocephalus*.

*^d^Intracranial hemorrhage resulting in NIHSS increase of ≥4*.

*^e^SITS-MOST definition (41)*.

*^f^ECASS-II definition (42)*.

Four studies reported unadjusted relative effects of antiplatelet agents on the risk of sICH (22, 32, 36, 39). Antiplatelet use was associated with a higher relative effect on sICH in two studies in which patients were on prior antiplatelet treatment (OR, 4.80; 95% CI, 1.77–13.02, and OR, 5.43; 95% CI, 1.46–20.13) (32, 36), and a neutral effect in the other studies in which patients received acute antiplatelet treatment in one and were on prior antiplatelet treatment in the other (OR, 0.92; 95% CI, 0.24–3.46, and OR, 0.81; 95% CI, 0.25–2.68) (22, 39). Only one study adjusted the estimate of the relative sICH risk, attributable to antiplatelet use, for prognostic factors (glucose level and baseline NIHSS), but not for prior comorbidity or reperfusion (36). The population of this study was heterogeneous, concerning patients who received IA plasminogen activator and/or MT. The absolute sICH risk was 13% among patients receiving prior antiplatelet treatment and 3% among patients who did not. Prior use of an antiplatelet agent was an independent risk factor for sICH (aOR, 8.03; 95% CI, 1.83–41.70).

Three studies reported unadjusted effect estimates of antiplatelet use on mortality and functional independence (22, 32, 39). The relative effect on mortality was neutral in two studies (OR, 0.75; 95% CI, 0.34–1.67, and OR, 0.97; 95% CI, 0.50–1.85) (22, 39) and higher in the other (OR, 2.46; 95% CI, 1.27–4.76) (32), when antiplatelet agents were used. In all studies, the effect on functional independency was neutral (OR, 0.61; 95% CI, 0.32–1.17, and OR, 0.54; 95% CI, 0.28–1.05, and OR, 1.11; 95% CI, 0.63–1.97).

The *post hoc* analysis of the Multicenter Randomized CLinical trial of Endovascular treatment for Acute ischemic stroke in the Netherlands (MR CLEAN) was the only study in which prior antiplatelet use was directly compared to no prior antiplatelet use in patients who underwent acute MT (32). Prior antiplatelet use was associated with a higher risk of sICH (OR, 4.80; 95% CI, 1.77–13.02) and mortality (OR, 2.46; 95% CI, 1.27–4.76). However, prior antiplatelet use did not interact with MT treatment effect and safety parameters like sICH. Moreover, among patients with successful recanalization, patients on prior antiplatelet use were twice as likely to have a favorable functional outcome (39 vs. 18%, *P*_interaction_ = 0.025). One other study that investigated the recanalization rate found that patients on prior antiplatelet treatment have higher odds for successful recanalization (39).

### Antiplatelet Use in Patients With Tandem Lesions

We identified eight cohort studies in which patients with tandem lesions—that required intracranial MT with or without combined emergency carotid artery stenting—received antiplatelet agents as mandatory protocol-based care to prevent stent occlusion (Table 3) (21, 23, 25, 28–30, 34, 35). Antithrombotic agents in these studies included eptifibatide, tirofiban, abciximab, acetylsalicylic acid, clopidogrel, and heparin, alone or in combination. The observed sICH risk in the included studies ranged from 0 to 17% (Table 4). Mortality ranged from 0 to 39% and functional independence from 29 to 70%. No relative effects on sICH, mortality, or functional independence were reported.

**Table 3:** Characteristics of included studies investigating patients with ischemic stroke caused by a “tandem lesions” who underwent acute MT with or without emergency extracranial carotid stenting, who received periprocedural antithrombotic drugs as protocol-based care.

Study characteristics		Population characteristics				Treatment characteristics					Study treatment	
			
Reference	Study design	*N*	Age	NIHSS at baseline	Occlusion location	IV tPA, *n*/*N* (%)	IA tPA, *n*/*N* (%)	MT, *n*/*N* (%)	Stenting, *n*/*N* (%)	Time from symptom onset to recanalization therapy (min)	Antithrombotic treatment when stent deployment was performed	Time from symptom onset to antithrombotic treatment^a^
Behme et al. (21)	Retrospective cohort	170	Median: 64	Median: 15	Anterior circulation	122/170 (72%)	0/170 (0%)	170/170 (100%)	170/170 (100%)	Median: 98	**Periprocedural**: Center A, loading dose of eptifibatide 180 μg/kg; Center B, loading dose of ASA 500 mg and clopidogrel 375 mg; Center C, loading dose of tirofiban (weight-adapted); Center D, loading dose of ASA 500 mg IV, plus 5,000 IU UFH or tirofiban **Postprocedural**: Center A, continuous infusion eptifibatide for the first 24 h, hereafter dual antiplatelet treatment (loading clopidogrel 300 mg and ASA 500 mg); Center B, continuation clopidogrel 75 mg/d and ASA 100mg/d for 3 months; Center C, continuous infusion of tirofiban for the first 24 h, hereafter loading 500 mg ASA and 300 mg clopidogrel, continuation with 75 mg/d clopidogrel and 100 m/d for 3 months; Center D, loading dose of clopidogrel 500 mg, hereafter ASA 100 mg/d and clopidogrel 75 mg/d for 3 months	Acute

Cohen et al. (23)	Retrospective cohort	24	Mean: 66	Median: 18 (14–28)	Anterior circulation	10/24 (42%)	0/24 (0%)	24/24 (100%)	24/24 (100%)	Mean: 198	**Periprocedural**: Loading dose of 2,500 IU UFH (after femoral access, and confirmation for the need of stent implantation), patients not on antiplatelet therapy received a loading dose of 300 mg ASA **Postprocedural**: Loading dose of clopidogrel 300 mg added to ASA use. Two months dual therapy (clopidogrel 75 mg/d plus ASA 100 mg/d)	Acute

Heck and Brown (25)	Retrospective cohort	23	Mean: 70	Median: 17 (9–25)	Anterior circulation	7/23: (30%)	0/23 (0%)	23/23 (100%)	23/23 (100%)	NR	**Periprocedural**: Loading dose of ASA 300 mg in all patients, 12 patients loading dose of abciximab 0.25 mg/kg **Postprocedural**: Loading dose of clopidogrel 600 mg if no abciximab was administered	NR

Lockau et al. (28)	Retrospective cohort	37	Mean: 63	Median: 17 (3–30)	Anterior circulation	20/37: (54%)	0/37 (0%)	37/37 (100%)	37/37 (100%)	NR	**Periprocedural**: Loading dose of tirofiban (weight adapted) **Postprocedural**: Continuous infusion of tirofiban for the first 24 h, after exclusion of hemorrhage loading dose of ASA 500 mg and clopidogrel 300 mg. Hereafter, ASA 100 mg/d and clopidogrel 75 mg/d for 3 months	Acute

Maurer et al. (30)	Retrospective cohort	43^b^	Mean: 68 (±13)	Mean: 13 (±5)	Anterior circulation	33/43 (77%)	20/43 (47%)	27/43 (63%)	39/43 (91%)	NR	**Periprocedural**: Loading dose of ASA (500 mg) and IV UFH bolus (5,000 IU) before stent placement **Postprocedural**: Loading dose of clopidogrel 600 mg or ticagrelor 180 mg	NR

Marnat et al. (29)	Retrospective cohort	20	Mean: 53	Mean: 18	Anterior circulation	15/20 (75%)	0/20 (0%)	20/20 (100%)	5/20 (25%)	Mean: 263	**Periprocedural**: Loading dose of ASA 500 mg **Postprocedural**: Local protocol	Acute + early

Rangel-Castilla et al. (34)	Retrospective cohort	45	Mean: 64	Mean: 14	Anterior circulation	15/45 (33%)	0/45 (0%)	45/45 (100%)	45/45 (100%)	Mean: 139	**Periprocedural**: Loading dose of ASA 650 mg and clopidogrel 600 mg or ticagrelor 180 mg. After confirmation of cervical occlusion heparinization at an activated coagulation time of ≥250 s **Postprocedural**: Local protocol at 24 h	Acute

Stampfl et al. (35)	Retrospective cohort	24	Mean: 67 (±10)	Median: 18 (15–22)	Anterior circulation	22/24 (92%)	0/24 (0%)	24/24 (100%)	24/24 (100%)	Mean: 230 (±131)	**Periprocedural**: 17 patients continuous infusion of tirofiban; 5 patients loading dose of ASA and clopidogrel and UFH; 2 patients (on prior antiplatelet) loading dose of UFH **Postprocedural**: Patients on tirofiban continuation for the first 24–48 h; all patients 100 mg/d ASA and clopidogrel 75 mg/d	Acute + early

*Characteristics of the included studies are presented by sample size (percentage), mean (SD), median (interquartile ranges), or by remarks*.

*ASA, acetylsalicylic acid; IA, intraarterial; IV, intravenous; MT, mechanical thrombectomy (by means of stent retriever or aspiration); NR, not reported; tPA, tissue plasminogen activator; UFH, unfractionated heparin*.

*^a^Time from symptom onset to antithrombotic treatment was divided into *acute* administration (<6 h) and *early* administration (6–24 h)*.

*^b^Not solely MT*.

**Table 4:** Outcomes of included studies investigating patients with ischemic stroke caused by a “tandem lesions” who underwent acute mechanical thrombectomy with or without emergency extracranial carotid stenting, who received periprocedural antithrombotic drugs as protocol-based care.

Reference	sICH, *n*/*N* (%)	Mortality, *n*/*N* (%)	mRS (0–2), *n*/*N* (%)
Behme et al. (21)	15/170 (9%)^a^	32/170 (19%)	62/170 (36%)
Cohen et al. (23)	0/24 (0%)^b^	2/24 (8%)	13/24 (54%)
Heck and Brown (25)	5/23 (2%)^c^	9/23 (39%)	12/23 (52%)
Lockau et al. (28)	4/37 (11%)^d^	7/37 (19%)	17/37 (46%)
Marnat et al. (29)	1/20 (5%)^a^	0/20 (0%)	14/20 (70%)
Maurer et al. (30)	5/43 (12%)^e^	9/43 (21%)	14/43 (33%)
Rangel-Castilla et al. (34)	2/45 (4%)^f^	5/45 (11%)	22/45 (49%)
Stampfl et al. (35)	4/24 (17%)^a^	4/24 (17%)	7/24 (29%)

*mRS, modified Rankin Scale; NIHSS, National Institutes of Health Stroke Scale; sICH, symptomatic intracranial hemorrhage*.

*^a^ECASS-II definition (42)*.

*^b^Intracranial hemorrhage resulting in NIHSS increase of ≥4 within 36 h*.

*^c^SITS-MOST definition (41)*.

*^d^Intracranial hemorrhage resulting in NIHSS increase of >4*.

*^e^No specific definition of sICH given*.

*^f^Intracranial hemorrhage resulting in NIHSS increase of ≥4 or death*.

### Thrombectomy and Heparin Use

Four studies investigated the periprocedural use of heparin (Table 5) (27, 33, 37, 38). Two studies were *post hoc* analyses of RCT data (33, 37), one was a cohort study (38), and one was an RCT investigating the efficacy of acute endovascular treatment, which could include periprocedural heparin use (27). All studies investigated the use of unfractionated heparin (UFH). Both anterior and posterior circulation occlusions were included in all studies. The administered heparin dose was reported in all studies and varied between 2,000 and 5,000 IU. Heparin administration was a part of standard care in one study (38) and left to the discretion of the interventionalist in three studies (27, 33, 37). The observed risk of sICH varied between 5 and 12%, mortality between 19 and 33%, and functional independence between 19 and 54% (Table 6).

**Table 5:** Characteristics of included studies investigating periprocedural heparin use in patients with ischemic stroke who underwent acute MT.

Study characteristics		Population characteristics				Recanalization therapy				Study treatment and contrast					
			
Reference	Study design	*N*	Age	NIHSS score at baseline	Occlusion location	IV tPA, *n*/*N* (%)	IA tPA, *n*/*N* (%)	MT, *n*/*N* (%)	Time from symptom onset to recanalization therapy (min)	Antithrombotic treatment	Indication for antithrombotic treatment	Time from symptom onset to antithrombotic treatment^a^	Treatment, *n*/*N* (%)	Control	Control, *n*/*N* (%)
**Cohort studies and ***post hoc*** analyses**															

Enomoto et al. (38)	Prospective cohort	704^b^	NR	NR	Anterior + posterior circulation	440/704 (63%)	123/704 (17%)	409/704 (58%)	NR	Standard UFH bolus of 3,000–5,000 IU, followed by 1,000 IU/h to maintain ACT (250–350 s)	Standard care	Acute + early	409/704^c^ (58%)	NR	NR

Nahab et al. (33)	*Post hoc* analysis on phase IIB RCT	51	Mean^d^: 75 (±10)	Mean^d^: 21 (±9)	Anterior + posterior circulation	18/51 (35%)	13/51 (25%)	51/51 (100%)	Mean^d^: 269 (±86)	UFH (median: 3,000 IU)	Discretion interventionalist	Acute + early	24/51 (41%)	No heparin	27/51 (53%)

Winningham et al. (37)	*Post hoc* analysis on phase III RCT	173	Mean: 68 (±14)	Median: 19 (15–21)	Anterior + posterior circulation	NR	96/173 (55%)	173/173 (100%)	<480	UFH (mean: 4,016 IU)	Discretion interventionalist	Acute + early	58/173 (34%)	No heparin	115/173 (66%)

**RCTs investigating effectiveness of endovascular strategies**															

Kidwell et al. (27)	Phase IIB RCT	64	Mean^e^: 66 (±13)	Median^e^: 16 (12–18)	Anterior circulation	44/64 (44%)	NR	64/64 (100%)	Mean^e^: 318 (±96)	Recommended UFH bolus of 2,000 IU, followed by 500 IU/h until end of procedure	Discretion interventionalist	Acute + early	NR	NR	NR

*Characteristics of the included studies are presented by sample size (percentage), mean (SDs), median (interquartile ranges), or by remarks*.

*IA, intraarterial; IU, international unit; IV, intravenous; MT, mechanical thrombectomy (by means of stent retrievers or aspiration); NIHSS, National Institutes of Health Stroke Scale; NR, not reported; RCT, randomized controlled trial; tPA, tissue plasminogen activator; UFH, unfractionated heparin*.

*^a^Time from symptom onset to antithrombotic treatment was divided in *acute* administration (<6 h) and *early* administration (6–24 h)*.

*^b^Not solely MT*.

*^c^Antithrombotic treatment was used in all patients in the MT group*.

*^d^Extracted data from the subgroup that received heparin*.

*^e^Extracted data from the population that received penumbral embolectomy*.

**Table 6:** Outcomes of included studies investigating periprocedural heparin use in patients with ischemic stroke who underwent acute mechanical thrombectomy.

Reference	sICH, *n*/*N* (%)	Mortality, *n*/*N* (%)	mRS (0–2), *n*/*N* (%)	sICH, OR (95%CI)	Mortality, OR (95%CI)	mRS (0–2), OR (95%CI)	sICH, aOR (95%CI)	Mortality, aOR (95%CI)	mRS (0–2), aOR (95%CI)
**Cohort studies and ***post hoc*** analyses**									
Enomoto et al. (38)	T = 20/409 (5%)^c^	NR	NR	NR	NR	NR	NR	NR	NR
Nahab et al. (33)	T = 2/24 (8%)C = 3/27 (11%)^d^	T = 8/24 (33%)C = 11/27 (41%)	T = 13/24 (54%)C = 8/27 (30%)	0.73 (0.11 - 4.77)	0.73 (0.23 - 2.28)	2.81 (0.89 - 8.88)	NR	NR	5.89 (1.34 - 25.92)^a^
Winningham et al. (37)	T = 7/58 (12%), C = 5/115 (4%)^d^	T = 17/58 (29%)C = 32/115 (28%)	T = 23/58 (40%)C = 30/115 (26%)	3.02 (0.91 - 9.97)	1.08 (0.54 - 2.16)	1.86 (0.95 - 3.64)	NR	NR	5.30 (1.70 - 16.48)^b^

**RCTs investigating effectiveness of endovascular strategies**									
Kidwell et al. (27)	T = 3/64 (5%)^e^	T = 12/64 (19%)	T = 12/64 (19%)	NR	NR	NR	NR	NR	NR

*C, control; CI, confidence interval; aOR, (adjusted) odds ratio; mRS, modified Rankin Scale; NIHSS, National Institutes of Health Stroke Scale; NR, not reported; OR, odds ratio; sICH, symptomatic intracranial hemorrhage; T, treated with heparin*.

*^a^Adjusted for age and final revascularization success in one study*.

*^b^Adjusted for intubation during procedure, postdevice TICI 2b–3, diabetes mellitus, baseline NIHSS score, study device (Trevo vs. Merci), time from symptom onset to arterial puncture (hours), and congestive heart failure in the other*.

*^a^SITS-MOST definition (41)*.

*^b^ECASS-II definition (42)*.

*^c^No specific definition of sICH given*.

Two studies reported an unadjusted effect estimate of heparin on the risk of sICH (33, 37). Both studies suggest that the effect of heparin use on sICH was neutral (8 vs. 11%; OR, 0.73; 95% CI, 0.11–4.77, and 12 vs. 4%; OR, 3.02; 95% CI, 0.91–9.97) (33, 37). Both studies also reported unadjusted effect estimates for mortality and functional independence. For the latter, also adjusted effects were provided. Both studies suggested that the effect on mortality is neutral (OR, 0.73; 95% CI, 0.23–2.28, and OR, 1.08; 95% CI, 0.54–2.16). After adjustment for prognostic factors [age and final revascularization success in one study (33), and intubation during procedure, postdevice TICI 2b–3, diabetes mellitus, baseline NIHSS score, study device (Trevo vs. Merci), time from symptom onset to arterial puncture (hours), and congestive heart failure in the other (37)], periprocedural heparin use was positively associated with functional independence in both studies (aOR, 5.89; 95% CI, 1.34–25.92, and aOR, 5.30; 95% CI, 1.70–16.48).

One study—which identified predictors for sICH—used a periprocedural loading dose of 3,000 to 5,000 IU UFH, followed by a continuous infusion of 1,000 IU per hour according to standard care (referred to as systemic heparinization) (38). The absolute risk of sICH was 5% in patients who underwent MT and received systemic heparinization. No relative effect of heparin on sICH was reported in this study, neither were mortality nor functional independence.

In the one RCT investigating the effectiveness of MT, periprocedural heparin use was left to the discretion of the treating interventionalist (27). When used, an IV dose of 2,000 IU UFH followed by a subsequent continuous infusion of 500 IU per hour until the end of the procedure was recommended for patients undergoing MT. The risk of sICH in the MT group was 5%. No relative effect on sICH was reported. Both mortality and functional independence occurred in 19% of the patients in this study, but relative effects were not provided.

### Thrombectomy and Antithrombotic Combination Use

One study investigated different antithrombotic combination treatments in the early phase (<24 h) after ischemic stroke (26). Patients had relatively mild anterior or posterior circulation occlusions with a median baseline NIHSS of 11. The early antithrombotic treatment consisted of antiplatelet, anticoagulant, and combined antiplatelet with anticoagulant treatments (Table 7). The sICH rate in this study was 3%, mortality 8%, and functional independence 56% (Table 8). In this heterogeneous treatment group, in which patients received IV plasminogen activator, IA plasminogen activator, and/or MT, early antithrombotic treatment was not associated with sICH compared to standard antithrombotic treatment after multivariable adjustment (OR 0.56, 95% CI: 0.35 to 2.10) (26). However, both the small group that actually received the combination therapy and the lack of subanalyses limit the ability to draw conclusions on combination antithrombotic treatments used during MT. This study is mentioned separately, because it did not report outcomes by separate antithombotic regimens.

**Table 7:** Characteristics of included studies investigating combined antithrombotic treatments use in patients with ischemic stroke who underwent acute MT.

Study characteristics		Population characteristics				Recanalization therapy				Study treatment and contrast					
			
Reference	Study design	*N*	Age	NIHSS score at baseline	Occlusion location	IV PA, *n*/*N* (%)	IA PA, *n*/*N*(%)	MT, *n*/*N* (%)	Time from symptom onset to treatment (h)	Antithrombotic treatment	Indication for antithrombotic treatment	Time from symptom onset to antithrombotic treatment^a^	*n*/*N* (%)	Control	*n*/*N* (%)
Jeong et al. (26)	Prospective cohort	456^b^	Mean: 68 (±13)	Median: 11 (6–18)	NR	285/456 (63%)	NR	297/456 (65%)	Median: 174 (102–468)	Antiplatelet monotherapy (ASA or clopidogrel) Antiplatelet dual therapy (ASA + clopidogrel or cilostazol) Anticoagulant (LMWH, UFH, dabigatran, rivaroxaban, warfarin) Antiplatelet with anticoagulant (LMWH, UFH, dabigatran)	Timing was based on individual physicians choice	Acute + early	456/456 (100%)	NR	NR

*Characteristics of the included studies are presented by sample size (percentage), mean (SDs), median (interquartile ranges), or by remarks*.

*ASA, acetylsalicylic acid; IA, intraarterial; IV, intravenous; LMWH, low-molecular-weight heparin; MT, mechanical thrombectomy (by means of stent retriever or aspiration); NR, not reported; UFH, unfractionated heparin*.

*^a^Time from symptom onset to antithrombotic treatment was divided into *acute* administration (<6 h) and *early* administration (6–24 h)*.

*^b^Not solely MT*.

**Table 8:** Outcomes of included studies investigating combined antithrombotic treatments use in patients with ischemic stroke who underwent acute mechanical thrombectomy.

Reference	sICH, *n*/*N* (%)	Mortality, *n*/*N* (%)	mRS (0–2), *n*/*N* (%)
Jeong et al. (26)	T = 15/456 (3%)^a^	T = 36/456 (8%)	T = 256/456 (56%)

*mRS, modified Rankin Scale; NIHSS, National Institutes of Health Stroke Scale; sICH, symptomatic intracranial hemorrhage; T, treated with antithrombotics*.

*^a^Intracranial hemorrhage resulting in NIHSS increase of ≥4*.

## Discussion

Based on the available literature, an increased sICH risk for both periprocedural administration of antiplatelet agents and heparin may be expected. Notwithstanding this higher risk of sICH, we found promising results of early antithrombotics regarding functional outcome in ischemic stroke patients undergoing MT. Future studies, especially RCTs, need to determine if the potentially higher sICH risk can be outweighed by improved functional outcome.

### Antiplatelet Agents

Most studies reported a small but noteworthy higher risk of sICH. Only one study performed multivariable adjustment, in which an aOR of 8.03 was found (36). However, the CI was wide (95% CI, 1.83–41.70), and there may have been residual confounding. Promising results on functional outcome were seen when patients were on prior antiplatelet treatment and a complete recanalization was established, as patients were twice as likely to have a favorable functional outcome (32). This analysis has not been done by the other included studies. Furthermore, the effect of adding antiplatelet agents may have a different result in patients who were treated with IV rtPA (15). However, none of the included studies performed this additional analysis. No further inference was possible.

Previous large randomized trials have investigated the isolated use of antiplatelet agents in general populations of patients with ischemic stroke (i.e., no endovascular treatment) (16, 43). In these studies, the absolute sICH risk associated with antiplatelet administration was approximately 1% when the treatment was initiated within 48 h from symptom onset. MT with or without prior IV tPA bears a sICH risk of 4.4%, ranging from 0 to 7.7% in the large trials (1). The MR CLEAN *post hoc* analysis had not found an interaction between antiplatelet agents and the effect of MT on functional outcome. Taken together, the risk of sICH in patients who undergo MT for ischemic stroke within 6 h and the risk of sICH contributable to antiplatelet agents, this expected risk of sICH is in line with the range from 6 to 17% presented in our review (1, 16).

On the whole, periprocedural use of antiplatelet agents may be a useful adjunct, albeit with a higher sICH risk.

### Heparin

Although at least one of the reported studies suggested that periprocedural heparin increased the risk of sICH (37), both studies that reported a relative effect of heparin on functional independence showed favorable results (33, 37). However, the true impact of adjunct heparin use remains difficult to determine in these observational studies. Substantial between-center variability in the use of periprocedural heparin exists. Indications varied from no heparin use, to use at the discretion of the interventionalist, and to standard care.

A large RCT has previously investigated the isolated effect of heparin treatment within 48 h in a general population of patients with ischemic stroke (i.e., no endovascular treatment), which resulted in an absolute sICH risk of 1.2% (16). Taken together with the sICH risk of MT, this is in line with the sICH range of 5–12% presented in our review (1, 16). This frequency of sICH is also comparable to the sICH risk in patients treated with acute systemic recombinant tPA in the NINDS and ECASS-III trials (44, 45). In the PROlyse (recombinant prourokinase) in Acute Cerebral Thromboembolism (PROACT) trial—the only randomized double-blind placebo-controlled trial of IA treatment—the use of heparin, at the outset (acute phase) of IA delivery of placebo or recombinant prourokinase (pro-UK), was a significant predictor of both recanalization efficacy and sICH frequency (46). That study set the heparin protocol for the PROACT-II study, in which heparin was administered in combination with recombinant pro-UK. In PROACT-II, both patients in the IA treatment arm and the control arm received heparin; 4,000 IU in total. A non-significant increase of 8% in sICH risk in the endovascular treatment arm compared to the control arm of the study was observed in the univariable analysis, but also an improvement in functional outcome just significant after stratification for stroke severity (40). Based on the available literature, the overall higher risk of sICH may be offset by the improved odds for a functional independence when heparin is used periprocedurally.

### Strengths and Limitations

In light of two other reviews describing periprocedural antithrombotic use in ischemic stroke management, the strength of this study is the specific focus on MT, the emphasis on safety, the performance of a thorough systematic literature search and the identification of studies not included in both other reviews (47, 48). Another strength is the structured reporting of data according to the PRISMA Statement.

A limitation of this study is that some studies investigating periprocedural antithrombotic use in patients with tandem lesions were not identified by the initial search. This was because these studies did not provide keywords related to antithrombotic treatment use. When we became aware of this finding, we managed this problem through an extensive bibliographic review of the included studies related to this topic. We discussed this issue with our biomedical information specialist, and due to heterogeneity among keywords used in these studies, an additional search was not feasible. It is possible that selection bias has occurred regarding this distinct pathology. On the whole, the risk of sICH seems acceptable in patients with tandem lesions, but the results of this subpopulation should be interpreted with caution, as the causal effects of previous ischemia, misery perfusion, and sudden reperfusion alongside that of antiplatelet treatment cannot be untwined. Because patients with tandem lesions constitute a distinct subpopulation with ischemic stroke, results may also be less generalizable to results in patients with intracranial occlusions only, despite a similar treatment effect of acute MT in these patients (21). However, since limited evidence is available on the safety of periprocedural antiplatelet use in ischemic stroke patients undergoing acute MT, these studies provide valuable information and could therefore not be omitted.

Other limitations were the wide heterogeneity of inclusion criteria, treatment characteristics, and outcome definitions among studies. We found that some studies included patients with posterior circulation occlusion. These patients have a very poor prognosis with high mortality rates (49). Inclusion of these patients could have interfered with the reported outcomes. Furthermore, recanalization therapy varied among included studies from solely MT to more heterogeneous groups that received IV plasminogen activators, IA plasminogen activators, and/or MT. As no distinction was made in some studies, data specifically concerning patients who underwent MT could not always be extracted. This could have blurred the actual effect of periprocedural antithrombotic use in MT. Also, the use of IV plasminogen activator and IA plasminogen activator could have masked the actual sICH risk attributable to the antithrombotics. We also observed that the indication for antithrombotic administration depended on standard care, the discretion of the interventionist, and comorbidities, which dictated prior use. Patients with prior antiplatelet and heparin use were *a priori* more likely to have higher odds for worse outcome than the control group (due to comorbidity or occurrence of re-occlusion)—implying confounding by indication—which may hamper the interpretation of the outcomes and effect estimates. Even though few studies performed a multivariable analysis to adjust for confounding factors, this does not exclude the possibility that residual confounding has influenced our findings. Interpretation of the results of our review has been hampered by missing data in most studies regarding example collateral status, infarct size, and underlying medical conditions for which antithrombotics were administered. Besides, we cannot rule out the possibility of publication bias. Moreover, we could not take dosing into account because of the limited number of studies reporting this. As we focused on periprocedural antiplatelet and heparin use, the effect of other antithrombotic drugs such as direct oral anticoagulants (DOACs) and coumarin derivatives remains unanswered. We chose not to include DOACs and coumarin derivatives as these drugs are not readily available to be administered in the acute phase. Besides, there is no evidence that DOACs and coumarin derivatives can restore microvascular obstruction. Furthermore, the time interval between symptom onset and start of antithrombotic treatment ranged from naught (prior use), through 0–6 h (studies administering the antithrombotic drugs in the *acute* phase during MT), to 6–24 h (studies postponing the antithrombotic treatment to the *early* postprocedural phase). Based on the experimental work, it seems likely that the *acute* use of specific antithrombotic agents could (I) decrease the incidence of sICH by avoiding the later stages of injury evolution and (II) potentially add to improvement of outcomes by preventing or limiting microvascular occlusion within the regions of ischemic injury (6, 7, 9). As the exact underlying pathway by which antithrombotics act—direct link between IMR and antithrombotics—was not in the scope of this review, this should be explored in future research. An example supporting the statement that especially the *acute* phase is of clinical relevance is the use of IV plasminogen activator in current practice. IV plasminogen activator seems safe when used within 4.5 h after stroke onset and improves functional outcome. However, extending this time window increases the risk of sICH significantly offsetting the beneficial effect (50). Possibly, as no clear distinction in time windows (i.e., *acute* or *early*) for antithrombotic treatment was made in most studies, the antithrombotic treatment effect may have been underestimated. Finally, sICH was defined according to various classifications, which makes it difficult to compare sICH risk among studies. Most studies elaborated on the exact sICH definition used (21–26, 28, 29, 31, 32, 34, 35, 38, 39). Most commonly, sICH was defined as neurologic deterioration with a 4 or more point increase in NIHSS score in combination with intracranial hemorrhage on imaging. Not all studies elaborated on the exact definition used. Therefore, heterogeneity among studies could have led to overestimation or underestimation of the actual risk. Due to the large variety in sample sizes and the heterogeneity between studies, a more in-depth exploration will not be helpful.

## Conclusion and Future Directions

Current evidence on periprocedural antiplatelet and heparin use in ischemic stroke patients undergoing acute MT relies on a limited number of *post hoc* analyses and cohort studies. Methodological limitations of these studies warrant cautious interpretation of the results. RCTs investigating the effect of periprocedural antithrombotic treatment in MT are lacking. Some observational studies report a slight increase in sICH risk, which may be acceptable because they also suggest a beneficial effect on functional outcome. Well-conducted phase III RCTs focusing on the acute use of antithrombotic agents alone and in combinations during MT are therefore required. MR CLEAN-MED (“Multicenter Randomized CLinical trial of Endovascular treatment for Acute ischemic stroke in the Netherlands; the effect of periprocedural MEDication: heparin, antiplatelet agents, both or neither”) is an ongoing phase III trial that investigates the effect of periprocedural intravenous use of aspirin and/or UFH on functional outcome of ischemic stroke patients undergoing MT (ISRCTN 76741621). We expect that this trial will provide better insights in the balance between potential risks and benefits of the use of these periprocedural antithrombotics for these patients.

## Author Contributions

RG, VC, and BR wrote the first draft of the manuscript and contributed to conception and design of the study; BR, DD, and AL supervised the study; all authors contributed to manuscript revision and read and approved the submitted version.

## Conflict of Interest Statement

RvdG has nothing to disclose. VC has nothing to disclose. GdZ has nothing to disclose. Erasmus MC received compensation from Stryker and Bracco Imaging Ltd for activities of DD and AL as a consultant. In addition, DD and AL are the recipients of unrestricted grants from Dutch Heart Foundation, Dutch Brain Foundation, Stryker and Penumbra for the conduct of trials on acute treatment for stroke. BR has nothing to disclose. RvdG, VC, DD, AL, and BR are investigators for the MR CLEAN-MED (ISRCTN 76741621).
