# Supplementary material for: Periprocedural Antithrombotic Treatment During Acute Mechanical Thrombectomy for Ischemic Stroke: A Systematic Review
Source: Front Neurol. 2018 Apr 16;9:238. doi: 10.3389/fneur.2018.00238 (PMC5911634; doi:10.3389/fneur.2018.00238)
Supplement: Supplementary file 1 [file data_sheet_1.PDF]

Supplementary Material

Periprocedural antithrombotic use during mechanical thrombectomy for acute ischemic stroke: a literature review

Rob A. van de Graaf, MD*; Vicky Chalos, MD; Gregory J. del Zoppo, MD, PhD; Aad van der Lugt, MD, PhD; Diederik W.J. Dippel, MD, PhD; Bob Roozenbeek, MD, PhD

*** Correspondence:** Rob A. van de Graaf, MD, Department of Neurology and Radiology, Erasmus MC, Room Ee-2240a, 3015 CE, Rotterdam, The Netherlands, [r.a.vandegraaf@erasmusmc.nl], Tel. +3110 704 42 06

Search strategy performed in March 2018

Embase.com (Embase incl. Medline): 487

('brain infarction'/de OR 'brain ischemia'/de OR 'brain stem infarction'/de OR 'cerebellum infarction'/de OR 'cerebrovascular accident'/exp OR 'brain embolism'/exp OR 'occlusive cerebrovascular disease'/exp OR 'anterior circulation stroke'/de OR (((brain* OR cerebr* OR cerebell* OR lacunar* OR intracran* OR vertebrobasil* OR hemispher* OR intracran* OR intracerebral OR infratentorial OR supratentorial OR vertebr* OR 'anterior-circulation') NEAR/3 (infarct* OR ischemi* OR ischaemi* OR embol* OR thromb* OR occlus* OR hypoxi* OR accident OR attack OR stroke*)) OR ((cerebrovascular* OR cva OR cvas OR stroke*) NEAR/3 (ischem* OR ischaem* OR thromb* OR embol*))):ab,ti) AND ('thrombectomy'/exp OR 'embolectomy'/de OR 'endovascular surgery'/de OR (thrombectom* OR embolectom* OR ((endovascular* OR 'intra-arterial' OR intraarterial) NEAR/3 (surger* OR treatment* OR procedur* OR therap*))):ab,ti) AND ('pretreatment'/de OR (early OR (within NEXT/2 hour*) OR pretreatment* OR periprocedur* OR preprocedur* OR perioperative* OR preoperative* OR antecedent* OR ((pre OR before OR prior OR acute OR peri OR pre OR during OR before) NEAR/3 (treatment* OR procedur* OR therap*)) OR prestroke OR 'pre-stroke' OR adjunctive):ab,ti) AND ('anticoagulant agent'/de/mj OR 'anticoagulant therapy'/de OR 'heparin derivative'/exp/mj OR 'antithrombocytic agent'/exp/mj OR (anticoagulant* OR antithrombocyt* OR antithrombotic* OR antiplatelet* OR ((anti) NEXT/1 (thrombotic* OR thrombocytic* OR coagulant*)) OR 'acetylsalicylic-acid' OR clopidogrel OR heparin* OR warfarin* OR ticlopidine OR (fibrinogen* NEAR/3 antagonis*) OR prasugrel* OR abciximab* OR enoxaparin* OR dipyridamole* OR ticagrelor* OR eptifibatide* OR ((platelet* OR 'Factor-Xa' OR 'Factor-X' OR thrombin*) NEAR/3 (inhibitor* OR antagonist* OR anti OR antiaggregat*)) OR antithrombin* OR (GP NEAR/3 (Iib OR IIIa) NEAR/3 inhibitor*)):ab,ti) NOT ([animals]/lim NOT [humans]/lim) NOT ('Conference Abstract' OR Letter OR Note OR Editorial)/it AND english:la

Medline Epub (Ovid): 523

(exp "Brain Infarction"/ OR "Brain Ischemia"/ OR "Stroke"/ OR exp "Intracranial Embolism and Thrombosis"/ OR (((brain* OR cerebr* OR cerebell* OR lacunar* OR intracran* OR vertebrobasil* OR hemispher* OR intracran* OR intracerebral OR infratentorial OR supratentorial OR vertebr* OR anterior-circulat*) ADJ3 (infarct* OR ischemi* OR ischaemi* OR embol* OR thromb* OR occlus* OR hypoxi* OR accident OR attack OR stroke*)) OR ((cerebrovascular* OR cerebr*-vascul* OR

cva OR cvas OR stroke*) ADJ3 (ischem* OR ischaem* OR thromb* OR embol*))).ab,ti,kf.) AND ("Thrombectomy"/ OR exp "Embolectomy"/ OR exp "Endovascular Procedures"/ OR (thrombectom* OR embolectom* OR ((endovascular* OR "intra-arterial" OR intraarterial) ADJ3 (surger* OR treatment* OR procedur* OR therap*))).ab,ti.) AND ((early OR (within ADJ2 hour*) OR pretreatment* OR periprocedur* OR preprocedur* OR perioperative* OR preoperative* OR antecedent* OR ((pre OR before OR prior OR acute OR peri OR pre OR during OR before) ADJ3 (treatment* OR procedur* OR therap*)) OR prestroke OR "pre-stroke" OR adjunctive).ab,ti,kf.) AND (exp "Anticoagulants"/ OR exp "Heparin"/ OR exp "Platelet Aggregation Inhibitors"/ OR "Ticagrelor".nm. OR (anticoagulant* OR antithrombocyt* OR antithrombotic* OR antiplatelet* OR ((anti) ADJ1 (thrombotic* OR thrombocytic* OR coagulant*)) OR "acetylsalicylic-acid" OR Aspirin* OR clopidogrel OR heparin* OR warfarin* OR ticlopidin* OR (fibrinogen* ADJ3 antagonis*) OR prasugrel* OR abciximab* OR enoxaparin* OR dipyridamole* OR ticagrelor* OR eptifibatide* OR ((platelet* OR "Factor-Xa" OR "Factor-X" OR thrombin*) ADJ3 (inhibitor* OR antagonist* OR anti OR antiaggregat*)) OR antithrombin* OR (GP ADJ3 (Iib OR IIIa) ADJ3 inhibitor*)).ab,ti,kf.) NOT (exp animals/ NOT humans/) NOT ((congresses OR letter OR editorial).pt.) AND english.lg.

Cochrane CENTRAL (trials): 33

((((brain* OR cerebr* OR cerebell* OR lacunar* OR intracran* OR vertebrobasil* OR hemispher* OR intracran* OR intracerebral OR infratentorial OR supratentorial OR vertebr* OR 'anterior-circulation') NEAR/3 (infarct* OR ischemi* OR ischaemi* OR embol* OR thromb* OR occlus* OR hypoxi* OR accident OR attack OR stroke*)) OR ((cerebrovascular* OR cva OR cvas OR stroke*) NEAR/3 (ischem* OR ischaem* OR thromb* OR embol*))):ab,ti) AND ((thrombectom* OR embolectom* OR ((endovascular* OR 'intra-arterial' OR intraarterial) NEAR/3 (surger* OR treatment* OR procedur* OR therap*))):ab,ti) AND ((early OR (within NEXT/2 hour*) OR pretreatment* OR periprocedur* OR preprocedur* OR perioperative* OR preoperative* OR antecedent* OR ((pre OR before OR prior OR acute OR peri OR pre OR during OR before) NEAR/3 (treatment* OR procedur* OR therap*)) OR prestroke OR 'pre-stroke' OR adjunctive):ab,ti) AND ((anticoagulant* OR antithrombocyt* OR antithrombotic* OR antiplatelet* OR ((anti) NEXT/1 (thrombotic* OR thrombocytic* OR coagulant*)) OR 'acetylsalicylic-acid' OR clopidogrel OR heparin* OR warfarin* OR ticlopidine OR (fibrinogen* NEAR/3 antagonis*) OR prasugrel* OR abciximab* OR enoxaparin* OR dipyridamole* OR ticagrelor* OR eptifibatide* OR ((platelet* OR 'Factor-Xa' OR 'Factor-X' OR thrombin*) NEAR/3 (inhibitor* OR antagonist* OR anti OR antiaggregat*)) OR antithrombin*):ab,ti)

Web of Science: 228

TS=(((((brain* OR cerebr* OR cerebell* OR lacunar* OR intracran* OR vertebrobasil* OR hemispher* OR intracran* OR intracerebral OR infratentorial OR supratentorial OR vertebr* OR "anterior-circulation") NEAR/2 (infarct* OR ischemi* OR ischaemi* OR embol* OR thromb* OR occlus* OR hypoxi* OR accident OR attack OR stroke*)) OR ((cerebrovascular* OR cva OR cvas OR stroke*) NEAR/2 (ischem* OR ischaem* OR thromb* OR embol*)))) AND ((thrombectom* OR embolectom* OR ((endovascular* OR "intra-arterial" OR intraarterial) NEAR/2 (surger* OR treatment* OR procedur* OR therap*)))) AND ((early OR (within NEAR/2 hour*) OR pretreatment* OR periprocedur* OR preprocedur* OR perioperative* OR preoperative* OR antecedent* OR ((pre OR before OR prior OR acute OR peri OR pre OR during OR before) NEAR/2 (treatment* OR procedur* OR therap*)) OR prestroke OR "pre-stroke" OR adjunctive)) AND ((anticoagulant* OR

antithrombocyt* OR antithrombotic* OR antiplatelet* OR ((anti) NEAR/1 (thrombotic* OR thrombocytic* OR coagulant*)) OR "acetylsalicylic-acid" OR clopidogrel OR heparin* OR warfarin* OR ticlopidine OR (fibrinogen* NEAR/2 antagonis*) OR prasugrel* OR abciximab* OR enoxaparin* OR dipyridamole* OR ticagrelor* OR eptifibatide* OR ((platelet* OR "Factor-Xa" OR "Factor-X" OR thrombin*) NEAR/2 (inhibitor* OR antagonist* OR anti OR antiaggregat*)) OR antithrombin*)) NOT ((animal* OR rat OR rats) NOT (human* OR patient*))) AND DT=Article AND LA=English
